# Post‐Axial Polydactyly and Postnatal Pulmonary Stenosis Observed With a *SPRED1* Pathogenic Variant

**DOI:** 10.1002/pd.6829

**Published:** 2025-05-28

**Authors:** Alexander Gibbs, Muriel Holder‐Espinasse, Vijaya Ramachandran, Natalie J. Chandler

**Affiliations:** ^1^ NHS North Thames Genomic Laboratory Hub Great Ormond Street Hospital for Children NHS Foundation Trust London UK; ^2^ Department of Clinical Genetics Guy's Hospital London UK

**Keywords:** Legius syndrome, NF1, post‐axial polydactyly, pulmonary stenosis, *SPRED1*

**Summary**

- What's already known about this topic?
  ◦ Heterozygous variants in *SPRED1* are associated with Legius syndrome, characterized by multiple *cafe‐au‐lait* spots and variable dysmorphic features.
  ◦ Post‐axial polydactyly is a rarely reported feature and cardiac abnormalities are not currently known to be associated with Legius syndrome.
- What does this study add?
  ◦ Further weight to the association between post‐axial polydactyly and Legius syndrome.
  ◦ Further evidence that a postnatal diagnosis of pulmonary stenosis is in keeping with Legius syndrome.

## 1 Introduction

A White British couple spontaneously conceived their first pregnancy. The female partner reported no medical history. However, her brother and mother (who had died of pancreatic cancer) both had abnormal areas of skin pigmentation and were suspected of having Neurofibromatosis Type 1 (NF1) without ever undergoing genetic testing.

## 2 Fetal Phenotype

Routine anatomy scan at 20 + 3 weeks' gestation was unremarkable except for the head circumference being on the 10th centile. An additional growth scan was performed at 29 + 1 week' gestation, identifying early onset fetal growth restriction: head circumference, femur length and estimated fetal weight all below the 2^nd^ centile. Lower limb bilateral post‐axial polydactyly was also detected (see Table 1A). Fetal dopplers were abnormal with raised uterine and umbilical artery pulsatility indices.

**TABLE 1A:** Clinical data.

Case	Parental details			Gestation at diagnosis	Phenotypes (HPO terms)	Obstetric history	Family history	Outcome
1	Maternal	Age	37	29 + 3	Polydactyly (HP: 0010442) Valvular pulmonary stenosis (HP:0034350)	Pre‐eclampsia	Undiagnosed skin pigmentation disorder	Delivery at 32 + 4 WG via Caesarean section
		Ethnicity	White British					
	Paternal	Age	39					
		Ethnicity	White British					

## 3 Diagnostic Method

Fetal DNA was extracted from cultured amniocytes. Initial QF‐PCR for common trisomies and microarray were unremarkable. Antenatal trio exome sequencing and analysis using a fetal anomaly gene panel was carried out as previously described [1].

## 4 Diagnostic Results and Interpretation

This identified a heterozygous maternally inherited likely pathogenic variant in *SPRED1*: NM_152594.3:c.1149_1152del p.(Gly385IlefsTer20) (see Table 1B). Monoallelic pathogenic variants in the *SPRED1* gene are known to cause Legius syndrome. The variant caused a frameshift resulting in a premature truncating codon within the last exon and is therefore expected to escape nonsense mediated decay. There are multiple truncating variants 3′ of this variant classified as pathogenic in the ClinVar database LOVD [2] and the variant is predicted to destroy the sprouty‐related domain necessary to anchor SPRED1 in the membrane (PVS1_strong). The variant itself had been classified as pathogenic by five different laboratories in the ClinVar database (Variation ID 646826) and has been reported in multiple individuals in the literature (PS4_moderate). The variant is present in three individuals in the gnomADv4.1 population database consistent with the variable expressivity associated with Legius syndrome.

**TABLE 1B:** Genetic findings.

Procedure (gest age)	Direct/culture?	Performed test	Secondary confirmatory test	Gene (name; REFSEQ)	Known disease (OMIM)	Variant	ACMG classification	Criteria applied	Inheritance & zygosity	Interpretation
29 + 3	Direct	Trio exome. Fetal anomalies panel applied	Sanger sequencing	*SPRED1* (NM_152594.3:c.1149_1152del)	Legius syndrome (611431)	c.1149_1152delp	4	PS4_moderatPVS1_strong	AD, maternal	Consistent with diagnosis
						(Gly385IlefsTer20)				
						Heterozygous				

## 5 Pregnancy Outcomes and Neonatal Findings

**FIGURE 1:**
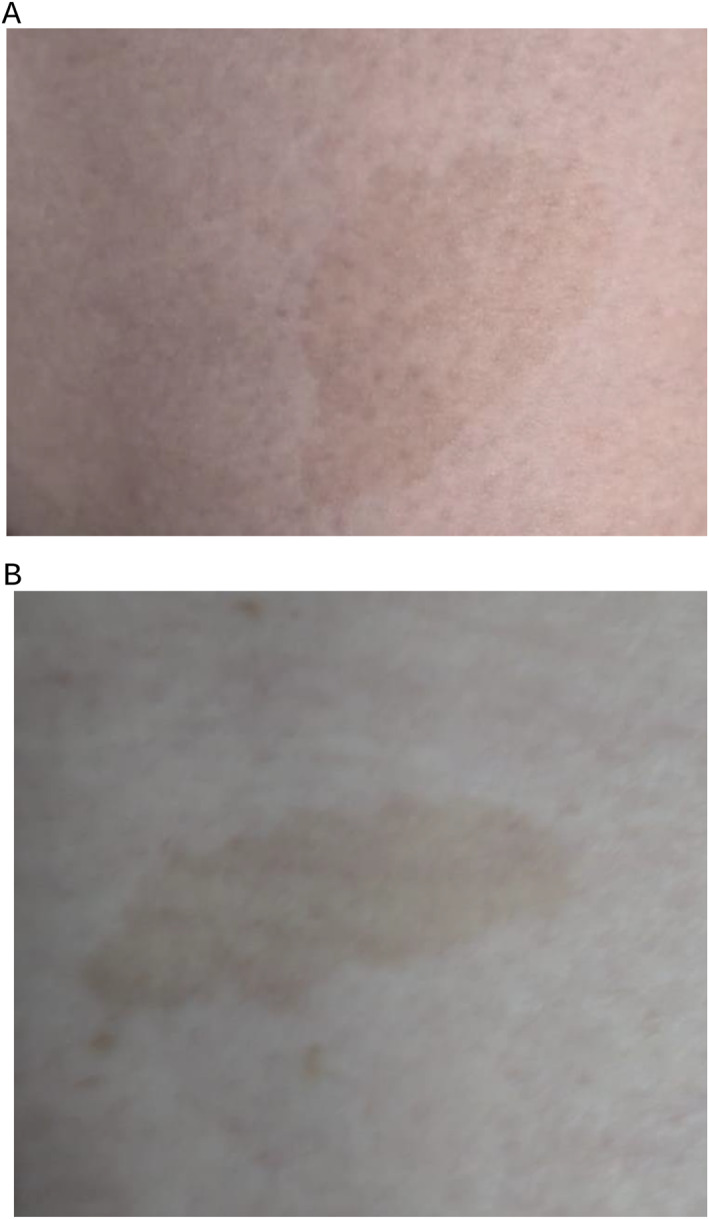
(A, B) Images of areas of skin pigmentation on mother. Key: (A) *Cafe‐au‐lait* spot on anterior lower abdomen of mother. (B) *Cafe‐au‐lait* spot on lower back of mother.

The mother developed rapidly progressing pre‐eclampsia (hypertension alongside the fetal growth restriction) during her third trimester, which evolved into HELLP syndrome. Consequently, a male baby was delivered by emergency Caesarean Section at 32 + 4 weeks' gestation. Birthweight was 1.27 kg. Post‐axial polydactyly was confirmed on both feet and identified in the hands. The baby was admitted to SCBU for 4 weeks, requiring Oxygen for 2 days and NG feeding for 3 weeks. Postnatally, the baby was diagnosed with pulmonary stenosis during an admission for bronchiolitis. At 12 months, there were no concerns regarding his development but growth remained below average: weight 2nd–9th centile, head circumference 0.4th–2nd centile. Detailed inspection of the mother elicited five *cafe‐au‐lait* spots on her abdomen and lower back (see Figure 1A,B), findings significantly less prominent than in her mother and brother.

## 6 Discussion

Legius syndrome (LGSS) is an autosomal dominant disorder displaying similarities with the more severe NF1 (NF1; 162200) [3]. Individuals with Legius syndrome typically have multiple *cafe‐au‐lait* spots, dysmorphic features such as hypertelorism, lipomas, and mild learning disabilities. NF1, caused by variants in the neurofibromin gene (613113), also involves c*afé‐au‐lait* spots. However, Legius syndrome is not associated with the neurofibromas or tumor predisposition seen with NF1.

Published literature already associates Legius syndrome with post‐axial polydactyly [4, 5]. Interestingly, Messiaen et al. reported a case of postaxial polydactyly with the same pathogenicity as seen in this fetus [4]. Another reported post‐axial polydactyly case was also born prematurely at 32 weeks' gestation due to placental dysfunction [5].

Associating the pulmonary stenosis observed in our case with Legius syndrome is more controversial given that prematurity is itself a risk factor for pulmonary stenosis. However, Messiaen et al. reported another case of Legius Syndrome where mild pulmonary artery stenosis was observed [4]. Furthermore, a publicly available database of patients with *SPRED1* variants records a child affected with a “heart murmur” [2], but further information on the nature of the cardiac condition was unavailable. The *SPRED1* gene encodes a negative regulator of the RAS‐MAPK pathway, like neurofibromin, and thus may be considered a RASopathy [3]. Other RASopathies have been associated with pulmonary stenosis, including NF1 [6]. This case provides further evidence that post‐axial polydactyly identified on fetal imaging can be indicative of a *SPRED1* variant, whilst also suggesting that cardiac abnormalities may need to be looked for with *SPRED1* variants, representing a new feature of Legius syndrome.

## Ethics Statement

The authors have nothing to report.

## Consent

Written parental consent was obtained to share the details of this case.

## Conflicts of Interest

The authors declare no conflicts of interest.

## Data Availability

The data that supports the findings of this report are available from the authors upon a reasonable request.
